# Insulin versus oral agents in the management of Cystic Fibrosis Related Diabetes: a case based study

**DOI:** 10.1186/1472-6823-6-4

**Published:** 2006-06-21

**Authors:** Gary M Onady, Leora J Langdon

**Affiliations:** 1 Wright State University School of Medicine, Medicine-Pediatrics Program, Suite 500 Elizabeth Place, Dayton, OH 45408, USA; 2 Children's Medical Center, Department of Pulmonology, One Children's Plaza, Dayton, OH 45404, USA

## Abstract

**Background:**

Insulin is the recommend therapeutic agent of choice for the management of Cystic Fibrosis Related Diabetes (CFRD), despite only sub-optimal reductions in glycemic control and increased morbidity and mortality reported by centers using this agent. The newer insulin sensitizing agents demonstrated to have anti-inflammatory mechanisms may provide an alternative management option for CFRD.

**Methods:**

A prospective case based therapeutic comparison between insulin, sulfonylurea, metformin and thiazolidinedione was observed over one decade with 20 CFRD patients diagnosed using American Diabetes Association guideline standards. Patients entering the study elected treatment based on risk and benefit information provided for treatment options. Patients receiving organ transplant or requiring combination diabetic medications were excluded from the study.

**Results:**

No statistical advantage was achieved regarding overall glycemic control for oral agents over insulin. Additional outcome measures including changes in weight, liver function testing and FEV_1 _were not statistically significant.

**Conclusion:**

Insulin alone may not be the only therapeutic option in managing CFRD. Oral hypoglycemic agents were equally effective in treating CFRD and may provide an alternative class of agents for patients reluctant in using insulin.

## Background

Diabetes mellitus has a 15–30% prevalence in cystic fibrosis (CF) patients [1]. Only 33% of diabetic CF patients are symptomatic for diabetes [2]. This subtle presentation of diabetes has resulted in the Cystic Fibrosis Foundation recommending screening guidelines for diabetes [3]. This guideline further recommends insulin as the therapeutic agent of choice in managing Cystic Fibrosis Related Diabetes (CFRD). Current recommendations for insulin are not always feasible for CFRD patients due to poor compliance and hypoglycemia. Diabetic diets utilized for type 1 and 2 diabetes conflict with the high fat and carbohydrate diet required to compensate for the malabsorption associated with the majority of CF patients. This carbohydrate load further stresses glucose tolerance in CF that is associated with both impaired insulin secretion and insulin resistance [4]. Increasing insulin resistance is correlated with progressive development of impaired glucose tolerance in CF [5,6]. Insulin deficiency progresses with increasing age, but is seldom absolute in CF and ketoacidosis is uncommon [7]. This disease process may therefore respond to medications used in the management of type 2 diabetes. Investigators testing this therapeutic potential reported the oral hypoglycemic agent, glipizide, effects on insulin secretion in CF diabetics [8]. Glipizide demonstrated a good response in early stages of disease, but overtly diabetic CF subjects had a poor response to glipizide alone. With the exception of this study, the medical literature has primarily focused on diabetic control with insulin therapy alone.

Inflammation may further impact insulin resistance in the CF patient. Oral insulin sensitizing agents with anti-inflammatory activity may provide a duel benefit in CFRD and perhaps serve as an alternative agent for managing diabetes. Metformin shares biguanide functional groups in common with protease inhibitors, and metformin has demonstrated protease inhibitor activity with exceptional potency at therapeutic concentrations [9]. The thiazolidinediones have additionally demonstrated a counter-regulatory protective leukotriene inflammatory effect [10]. These agents have not been previously evaluated for CFRD, secondary to concern of metabolic acidosis complicated by metformin and the potential of hepatic toxicity with thiazolidinediones [11].

This study reports the first clinical outcome for insulin sensitizing agents with potential anti-inflammatory activity in the management of CFRD. The oral agents reviewed above are compared to the insulin treatment group for each clinical outcome. Clinical outcome measures followed changes in glycosylated hemoglobin (HbA_1c_), weight, forced expiratory volulme at 1 second (FEV_1_), and alanine aminotransferase (ALT) for the duration of treatment for each group.

## Methods

Cystic Fibrosis Related Diabetes was diagnosed in 24 patients during a 10 year span from January 1992 through December 2002 at the Dayton Adult Cystic Fibrosis Program. Three CFRD patients receiving lung transplant were excluded from this study secondary to immunosuppressive therapy that included prednisone. A patient on combination diabetic therapy during the study period was also excluded. A total of 20 CFRD patients over this 10 year period remained for this prospective study.

Patients elected treatment after informed consent was provided for all management options based on the benefit of oral agents approved for use in type 2 diabetes mellitus but with explicit concerns regarding risks of these agents in context of the chronic illness and morbidity associated with CF. The patients were further instructed that the Cystic Fibrosis Foundation recommended the use of insulin as the preferred method in treating CFRD. Both hospital and university institutional review boards approved of this study protocol. Eight patients initially chose insulin, 5 patients sulfonylureas, 4 patients metformin (a biguanide), and 3 patients thiazolidinediones.

The diagnosis of diabetes was established based on the most current American Diabetes Association guidelines during the decade span of the investigation based on a minimum of two threshold fasting blood glucose and/or random blood glucose values with symptoms. Weights were used over BMI in this adult population as height data remained constant. All weights were obtained standing, without shoes on a balance scale calibrated yearly.

Patients were initiated on all oral agents starting with the lowest dose recommended based on standard pharmaceutical recommendations. Patients were evaluated at three month intervals at which time HbA1c levels were obtained. All patients with HbA1c >7 had a dose increased to the next recommended incremental level and followed up 3 months later. Once an optimal HbA1c was achieved, follow-up was spaced to 6 month intervals with HbA1c levels drawn and adjusted around the HBA1c >7 parameter. Insulin doses used consisted of split dosing 70/30 NPH/R ranging from 0.3 – 0.8 Units/kg. The oral medication dosing range required to achieve optimal control expressed as a percentage of the highest recommend dose was: sulfonylureas, 0.125 – 0.87; thiazolidinediones, 0.125 to 1.00; and metformin, 0.39 – 1.00. Compliance was followed by timing of prescription renewal with minimal differences identified between treatment groups.

### Statistical analyses

All continuous data are presented as the mean ± standard deviation (SD). Comparisons between groups on baseline data (age, weight, FEV_1_, and HbA_1c_) and treatment duration were made with one-way analysis of variance (ANOVA), or Welch ANOVA if the variances within groups were not equal. The changes in clinical variables from the start to the end of the treatment period were determined, and then were converted to changes/year to adjust for differences in the duration of treatment. Changes/year was compared between groups with either one-way ANOVA or Welch ANOVA. P values less than 0.05 were considered statistically significant. Chi-squared testing for nominal mortality data from the GraphPad web based statistical program.

## Results

All patient clinical profiles at the start of the study year, 1992, or at the time of CFRD diagnosis are summarized in Table 1 and their clinical data during the study until death or end of 2002 is graphed in the Figure. Baseline clinical data for patients are grouped by the specific agent used to manage CFRD and is summarized in Table 2. Data reported in Table 3 reflect the variation over the course of study from the baseline parameters identified in Table 2.

**Table 1:** Clinical profile of patients followed at start of study

**Pt**	**Age (yrs)**	**Wt (kg)**	**FEV_1 _(%)**	**% HbA_1c _at Dx**	**Rx Course (yrs)**	**Clinical Data**
I_1_	28	65.8	56	5.5	4	PI, Lupus^N^, Deceased
I_2_	31	65.3	44	14.3	2	PI, Deceased
I_3_	13	41.9	57	9.3	4	PS
I_4_	33	54.3	18	10.0	2	PI, Deceased, RI*
I_5_	14	49.0	99	10.9	8	PI
I_6_	44	81.5	30	9.2	5	PI, Cor P, HTN, BPH
I_7_	24	53.0	57	7.3	3	PI, AIHS, RI
I_8_	21	70.5	93	9.8	10	PI
						
S_1_	29	75.5	71	7.1	1	PI
S_2_	17	33.6	33	8.1	2	PI, Deceased
S_3_	25	73.9	48	7.8	4	PI, Deceased
S_4_	33	97.5	83	6.3	1	PI, Bipolar, HTN
S_5_	18	49.7	30	6.6	1	PI, Deceased
						
M_1_I_4_	35	51.5	17	9.7	1	PI
M_2_	24	71.1	117	5.4	2	PI
M_3_I_5_	22	68.5	68	9.6	2	PI
M_4_	19	70.0	100	6.1	3	PI
M_5_	22	67.7	88	13.3	3	PI, Hepatic Cirrhosis
M_6_	15	41.8	85	8.9	5	PI
						
T_1_	29	58.0	47	13.3	2	PI
T_2_	20	44.4	23	5.1	1	PI
T_3_I_6_	49	85.5	29	9.2	2	PI, Cor P, HTN, BPH
T_4_I_7_	32	55.7	65	7.3	2	PI, AIHS, RI
T_5_S_4_	34	96.5	92	6.3	1	PI, Bipolar, HTN
T_6_	40	54.0	56	9.6	2	PI, Lupus, RI ABPA

Pt, patient (subscripts identify patient number, e.g. M_1_I_4 _was the first patient started on metformin and the fourth patient started on insulin); Rx Course (Yrs), represents the number of years that a patient received consecutive therapy with a specific class of agent; Mon, months; I, insulin; S, sulfonylurea; M, metformin; T, thiazolidinediones, FEV_1_, forced expiratory volume at 1 second; HbA_1c_, percent glycosylated hemoglobin; PI, pancreatic insufficient; PS, pancreatic sufficient; Lupus^N^, lupus with nephritis; RI, renal insufficiency; Cor P, cor pulmonale; HTN, hypertension; BPH, benign prostatic hypertrophy; AIHS, autoimmune hypersplenism; ABPS, allergic bronchopulmonary aspergillosis. *Patient was switched back to insulin when diagnosed with renal insufficiency.

**Table 2:** Baseline clinical data

**Variable Mean ± SD (Range)**	**Insulin (n = 8)**	**Sulfonylurea (n = 5)**	**Metformin (n = 6)**	**Thiazoli-dinediones (n = 6)**	**P Value**
Age (Yrs)	26.0 ± 10.3 (13 – 44)	24.4 ± 6.9 (17 – 33)	22.8 ± 6.7 (15 – 35)	34.0 ± 9.9 (20 – 49)	0.166
ALT (U/L)	65.1 ± 24.1 (25 – 99)	42.0 ± 26.5 (14 – 73)	68.3 ± 28.1 (28 – 107	67.4 ± 8.4 (55 – 76) (n = 5)	0.250
FEV_1 _(%)	56.8 ± 28.0 (18 – 99)	53.0 ± 23.3 (30 – 83)	79.2 ± 34.5 (17 – 117)	52.0 ± 25.2 (3 – 92)	0.329
Weight (kg)	60.2 ± 12.9 (41.9 – 81.5)	66.0 ± 24.8 (33.6 – 97.5)	61.8 ± 12.2 (41.8 – 71.1)	65.7 ± 20.4 (44.4 – 96.5)	0.911
HbA_1c_	9.5 ± 2.6 (5.5 – 14.3)	7.2 ± 0.8 (6.3 – 8.1)	8.8 ± 2.8 (5.4 – 13.3)	8.0 ± 3.1 (5.1 – 13.3)	0.408
Treatment Duration (Yrs)	4.8 ± 2.9 (2 – 10)	1.8 ± 1.3 (1 – 4)	2.7 ± 1.4 (1 – 5)	1.8 ± 0.4 (1 – 2)	0.093

SD, standard deviation; Yrs, years; ALT, alanine aminotransferase; FEV_1_, forced expiratory volume at 1 second; HbA_1c_, percent glycosylated hemoglobin. P values for Age, ALT, FEV_1_, Weight, and HbA_1c _are from one-way analysis of variance (ANOVA); the P value for Treatment Duration is from Welch ANOVA for unequal group variances.

**Table 3:** Clinical data variance over time

**Variable Mean ± SD (Range)**	**Insulin (n = 8)**	**Sulfonylurea (n = 5)**	**Metformin (n = 6)**	**Thiazoli-dinediones (n = 6)**	**P Value**
ALT (U/L) Change/Yr	1.8 ± 3.0 (-2.0 – 7.8)	4.7 ± 8.6 (-2.5 – 19.0)	-2.8 ± 22.0 (-41.5 – 26.5)	-1.1 ± 7.7 (-11.0 – 9.5) (n = 5)	0.757
FEV_1 _(%) Change/Yr	-0.3 ± 3.3 (-3.9 – 6.3)	1.4 ± 9.6 (-9.0 – 13.0)	-1.2 ± 3.2 (-7.0 – 1.5)	5.2 ± 7.8 (-2.5 – 20.0)	0.422
Weight (kg) Change/Yr	1.5 ± 2.2 (-1.2 – 6.3)	0.5 ± 2.2 (-2.4 – 2.7)	1.8 ± 3.9 (-3.3 – 8.5)	4.3 ± 6.0 (0.3 – 16.0)	0.384
HbA_1c _Change/Yr	-0.3 ± 0.2 (-0.5 – 0.1)	-0.8 ± 0.7 (-1.8 – -0.1)	-1.1 ± 1.4 (-3.2 – 0.6)	-0.6 ± 1.3 (-3.2 – 0.5)	0.283

SD, standard deviation; Yr, year; ALT, alanine aminotransferase; FEV_1_, forced expiratory volume at 1 second; HbA_1c_, percent glycosylated hemoglobin. P values for ALT and Weight are from one-way analysis of variance (ANOVA); P values for FEV_1 _and HbA_1c _are from Welch ANOVA for unequal group variances.

Seven individuals (35%) had HbA_1c _levels < 7% at the time of diagnosis. All patients tolerated initial therapy well, and no patient changed medical management due to complications reported for these agents. Four patients chose to discontinue insulin therapy for an oral agent secondary to inadequate glycosylated hemoglobin control of 7.0% while using insulin. Patient M_1_I_4 _weaned off 60 units of insulin per day with the best HbA_1c _control at 9.7% to metformin therapy achieving an averaged HbA_1c _of 6.5%. This individual was the only patient in the metformin group with an FEV_1 _< 60% (refer to Figure), and was insistent on using this agent despite strong advise to select another treatment option. Patient T_5_S_4 _was well controlled on a sulfonylurea, but requested to switch to a thiazolidinedione.

Mortality rates from our study were highest among patients in the sulfonylurea group (60%); followed by the insulin group (37%); with no deaths observed from the biguanide and thiazolidinedione treatment groups. Death rates between treatment groups were not statistically significant; P = 0.062. Sixty patients had been followed in this adult center during this ten year period with a mortality rate of 23% observed in non-diabetics and 38% in diabetics, of which only a third were female, for a 1.7 increased diabetic mortality risk which is within the range reported by other centers [12,13]. To date, only one patient, T_6_, has been identified with diabetic complications. That patient came from the thiazolidinedione treatment group and followed for systemic lupus. A nephropathy developed 18 months after the diagnosis of CFRD was made, with renal biopsy indicating diabetic nephropathy. There have been no further reports of abnormal urine microalbumin measures or retinal examinations indicating microvascular disease in our patients.

## Discussion

Results demonstrate no statistical advantage of one treatment option over another in achieving overall glycemic control. The number of patients in the various treatment groups was most likely too small to achieve statistical significance. More aggressive overall glycemic control for CFRD may be necessary based on recent reports that HbA_1c _underestimates a true glycemic index in CF patients [14]. A reduced life span of red blood cells has recently been reported through personal communication in cystic fibrosis patients by researches in Houston, Texas, which may reflect this underestimate of glycemic control. Studies from the CF literature further demonstrate insulin achieves only sub-optimal glycemic control based on HbA_1c _outcomes, which is a significant concern when placed in context with the data demonstrating that HbA_1c _underestimates glycemic control. A retrospective study out of Cleveland, Ohio, evaluated 22 patients on a flexible meal-planning system targeting insulin boluses titrated to each meal to control postprandial blood glucose excursions and report glycosylated hemoglobin reductions from 11.3 to 8.1% [15]. A prospective study from Paris, France, followed 14 patients early in the diabetic course on insulin therapy finding glycosylated hemoglobin values ranging from 6.6 to 7.8% [16]. A study conducted in Houston, Texas compared subcutaneous insulin injections versus pump infusion demonstrated improved glycemic control on the insulin pump in lowering HbA_1c _values from 8.6 to 7.3% [17].

Morbidity and mortality data reported by centers using insulin as standard management protocol provide concern regarding optimizing clinical outcomes for CFRD. Microvascular disease is reported in 23% of diabetic CF patients [18] compared to 18% [19] in the non-CF diabetic population. The median age of survival for CFRD patients is reported at 35.6 years compared to 47 years for non-diabetics with CFRD females having a 7-fold mortality rate [20]. Significant mortality relative risks of 1.7 [12] and 2.8 [13] have been reported in CFRD patients over non-diabetic CF patients at two other centers.

Our center diabetic outcomes observed no adverse side effects from oral agents during our decade experience of treating CFRD. Caution must still be provided regarding the potential side effects of using the newer oral agents in managing CF diabetics, with the biguanide class presenting the most concerning risk of metabolic acidosis in the CF patient, and therefore should not be offered to the 30% of CF patients that have well documented liver disease by the time they reach adulthood. This study has furthermore identified four patients (20%) treated with renal insufficiency, in which only the one case mentioned above was attributed to diabetic nephropathy. Renal insufficiency is an absolute contraindication to the use of metformin and therefore, renal function must be followed closely in all CF patients as they approach their fourth and fifth decade of life. Likewise, this class of agent should be utilized only in patients with good to moderate pulmonary function. A recent study reported the safety of metformin in 91 randomized patients by intention-to-treat analysis with chronic obstructive pulmonary disease (COPD). Lactic acid values did not differ in the groups on or off metformin and correlated only with serum creatinine and body mass index. Mortality data were identical in the two groups and they conclude that there is no apparent reason that COPD patients should discontinue metformin [21]. Additionally in a systematic review of 194 randomized control studies of metformin with other diabetic agents, no fatal or nonfatal lactic acidosis events were reported, with Poisson statistics at 95% confidence intervals estimating lactic acidosis incidence at 8.1 and 9.9 cases per 100,000 [22].

A remarkable outcome from this center experience is the observed 1.8 kg weight gain for patients taking metformin. Weight loss, is typically reported for the type 2 diabetic, and this outcome would not be tolerated in most cystic fibrosis patients. The largest weight gain observed by a patient in this group was a very respectable 27%. One patient did lose 3% and another lost 7% of weight from this treatment group which explains the large range in the standard deviation of weight for this class. Neither of the patients losing weight demonstrated a loss of pulmonary function, with the patient losing 7% weight actually demonstrating an FEV_1 _gain of 4% per year. The lower pulmonary co-morbidity in the metformin group over other groups may have influenced the higher weight gain observed in this group. While this reduces the matching between groups, statistical significance was not observed over the insulin treatment group. Reasons behind a positive weight gain on metformin may relate to the cystic fibrosis patient having a higher rate of protein breakdown than non-CF patients [23]. Suppression of proteolysis by protease inhibitor activity of metformin [9] may both optimize overall nitrogen balance as well as inhibit insulin degradation. There have not been any deaths from our diabetic population maintained on metformin during our decade long experience in treating CFRD. Liver enzymes were least affected in this group as well as in the thiazolidinedione treatment group which also demonstrated a net decrease in liver enzyme changes during the course of therapy, and both were within the range of change observed with the other agents used in managing diabetes.

This study is limited by the small number of patients treated in a non-randomized, un-blinded fashion under each therapeutic grouping. Additional bias is potentially introduced by including baseline data of patients entering the study diagnosed with CFRD prior to prospective data gathering that was initiated in 1992, at which point all patient data was prospectively reviewed in this cohort study. Pulmonary treatment further evolved throughout the duration of this review in relation to standards of airway clearance utilized and aerosolized maintenance therapies that had become available to these patients.

## Conclusion

Our CF center has adopted a rational approach to managing CFRD based on this observational experience.

1) Insulin appears to provide adequate response to patients with significant lung disease (FEV_1 _< 60%), and is likely the agent of choice to initiate management under any inpatient setting.

2) After initial response to insulin, consideration should be given to add a sulfonylurea or thiazolidinedione for patients with stable liver functions, in attempt to wean off insulin.

3) In the patient with significant diabetic onset with HbA_1c _> 7 and relatively preserved pulmonary function (FEV_1 _> 60%) with no documented liver disease, metformin can be used for initial management on an outpatient basis. Close monitoring of metabolic profile, renal, and hepatic function at quarterly intervals would be optimal.

4) The thiazolidinedione class is an alternative agent for outpatient management, particularly for the patient with FEV_1 _close to or approaching 60% predicted. In this situation liver function testing should be monitored quarterly at onset of therapy.

Our findings suggest that insulin may not be the most beneficial therapeutic agent for the management of CFRD. Oral agents that include the insulin sensitizing agents appear to be safe and as effective as insulin. Larger randomized control trials between insulin and the biguanide or thiazolidinedione class of agents with potential anti-inflammatory activity should be considered as alternative therapy to insulin, especially in light of consistently poor clinical outcomes reported for insulin therapy in the CF patient population.

## Competing interests

The author(s) declare that they have no competing interests.

## Authors' contributions

Gary M. Onady was responsible for study design, calculation of statistical outcomes and drafting of the manuscript. Leora J. Langdon was responsible for collection of the majority of patient data and to some of the informational content presented in the manuscript.

**Figure 1:**
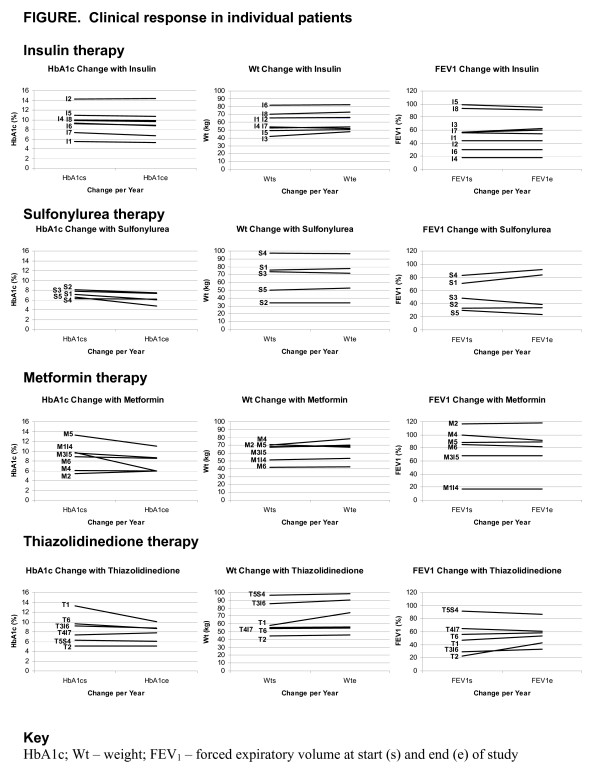
Clinical response in individual patients.

## Pre-publication history

The pre-publication history for this paper can be accessed here:



## References

[B1] Hayes FJ, O'Brien C, FitzGerald MX, McKenna MS (1994). Diabetes mellitus in an adult cystic fibrosis population. Ir Med J.

[B2] Lanng S, Hansen A, Thorsteinsson B, Nerup J, Doch C (1995). Glucose tolerance in patients with cystic fibrosis: five year prospective study. BMJ.

[B3] Aitken ML (1997). Clinical Practice Guidelines for Cystic Fibrosis, Appendix V – Screening for Diabetes Mellitus in Patients with Cystic Fibrosis. Appendix V.

[B4] Moran A, Diem J, Klein DF, Levitt MD, Robertson RP (1991). Pancreatic endocrine function in cystic fibrosis. J Pediatr.

[B5] Moran A, Pyzdrowski KL, Witreb J, Kagn BB, Smith SA, Adams KS, Seaquist ER (1994). Insulin sensitivity in cystic fibrosis. Diabetes.

[B6] Austin A, Kalhan SC, Orenstein D, Nixon P, Arslanian S (1994). Roles of insulin resistance and β-cell dysfunction in the pathogenesis of glucose intolerance in cystic fibrosis. J Clin Endo Metab.

[B7] Rodman HM, Doershuk DF, Roland JM (1986). The interaction of two diseases: diabetes mellitus and cystic fibrosis. Medicine.

[B8] Culler FL, McKean LP, Buchanan CN, Caplan DB, Meacham LR (1994). Glipizide treatment of patients with cystic fibrosis and impaired glucose tolerance. J Pediatr Gastroenterol Nutr.

[B9] Sweeney D, Raymer ML, Lockwood TD (2003). Antidiabetic and antimalarial biguanide drugs are metal-interactive antiproteolytic agents. Biochem Pharmacol.

[B10] Yamashita M, Kushihara M, Hirasawa N, Takasaki W, Takahagi H, Takayanagi M, Ohuchi K (2000). Inhibition by troglitazone of the antigen-induced production of leukotrienes in immunoglobulin E-sensitized RBL-2H3 cells. Br J Pharmacol.

[B11] Hardin DS, Moran A (1999). Diabetes mellitus in cystic fibrosis. Endocrin Metabolism Clinics of North Am.

[B12] Doershuk CF, Schluchter MD, Konstan MW A case-controlled study of the effect of diabetes mellitus on survival and pulmonary function in cystic fibrosis [abstract]. Fifteenth Annual North American Cystic Fibrosis Conference, Orlando Marriott World Center, Orlando, Florida, October 25–28, 2001.

[B13] Lanng S, Throsteinsson B, Koch C CF-related diabetes: a ten-year prospective study of diagnostic criteria, prevalence, late diabetic complications and mortality[abstract]. Fifteenth Annual North American Cystic Fibrosis Conference, Orlando Marriott World Center, Orlando, Florida, October 25–28, 2001.

[B14] Dobson L, Sheldon CD, Hattersley (2004). Conventional measures underestimate glycaemia in cystic fobrosis patients. Diabetic Med.

[B15] Hayes DR, Sheehan JP, Ulchaker MM, Rebar JM (1994). Management dilemmas in the individual with cystic fibrosis and diabetes. J Am Diet Assoc.

[B16] Rolan MA, Benali K, Munck A, Navarro J, Clement A, Tubiana-Rufi N, Czernichow P, Polak M (2001). Cystic fibrosis-related diabetes mellitus: clinical impact of prediabetes and effects of insulin therapy. Acta Paediatr.

[B17] Hardin DS, Rice J, Hale K Use of the insulin pump to treat CF related diabetes. Abstract 483, Fifteenth Annual North American Cystic Fibrosis Conference, Orlando Marriott World Center, Orlando, Florida, October 25–28, 2001.

[B18] Landers A, Mathalone B, Gyi KM, Hodson ME Diabetic retinopathy in patients with cystic fibrosis related diabetes (CFRD) [abstract]. Eleventh Annual International Cystic Fibrosis Conference, Nashville Tennessee, October 26th, 1997.

[B19] Dagogo-Jack S, Santiago JV (1997). Pathophysiology of type 2 diabetes and modes of action of therapeutic interventions. Arch Intern Med.

[B20] Milla CE, Moran AM Females with CF related diabetes are at higher risk of early mortality[abstract]. Seventeenth Annual North American Cystic Fibrosis Conference, Anaheim Convention Center, Anaheim, California, October 16–19, 2003.

[B21] Rachmani R, Slavachevski I, Levi Z, Zadok B, Kedar Y, Ravid M (2002). Metformin in patients with type 2 diabetes mellitus: reconsideration of traditional contraindications. Eur J Intern Med.

[B22] Salpeter SR, Greyber E, Pasternak GA, Salpeter EE (2003). Risk of fatal and nonfatal lactic acidosis with metformin use in type 2 diabetes mellitus: systematic review and meta-analysis. Arch Intern Med.

[B23] Hardin DS, LeBlanc A, Lukenbaugh S, Para L, Seilheimer DK (1998). Proteolysis associated with insulin resistance in cystic fibrosis. Pediatrics.

